# Microbiome in Hidradenitis Suppurativa—What We Know and Where We Are Heading

**DOI:** 10.3390/ijms231911280

**Published:** 2022-09-24

**Authors:** Zuzanna Świerczewska, Miłosz Lewandowski, Agnieszka Surowiecka, Wioletta Barańska-Rybak

**Affiliations:** 1 Department of Dermatology, Venereology and Allergology, Faculty of Medicine, Medical University of Gdansk, Smoluchowskiego 17, 80-214 Gdansk, Poland; 2 Faculty of Medicine, Medical University of Gdansk, Marii Skłodowskiej-Curie 3a, 80-210 Gdansk, Poland; 3 East Center of Burns Treatment and Reconstructive Surgery, District Hospital in Łęczna, ul. Krasnystawska 52, 21-010 Łęczna, Poland

**Keywords:** hidradenitis suppurativa, acne inversa, interleukins, microbiome, antimicrobial peptides

## Abstract

Recently, interest in the microbiome of cutaneous diseases has increased tremendously. Of particular interest is the gut-brain-skin axis proposed by Stokes and Pillsbury in 1930. The microbiome has been suggested in the pathogenesis of hidradenitis suppurativa, however the link between the commensals and the host is yet to be established. Across all studies, the increased abundance of *Porphyromonas*, *Peptoniphilus,* and *Prevotella* spp., and a loss of skin commensal species, such as *Cutibacterium* in HS lesions, is a consistent finding. The role of gut and blood microbiome in hidradenitis suppurativa has not been fully elucidated. According to studies, the main link with the intestine is based on the increased risk of developing Crohn’s disease and ulcerative colitis, however, further research is highly needed in this area. Lifestyle, dietary approaches, and probiotics all seem to influence the microbiome, hence being a promising modality as adjuvant therapy. The aim of this review was to present the latest reports in the field of research on skin, blood, and gut microbiome in terms of hidradenitis suppurativa.

## 1. Introduction

Hidradenitis suppurativa (HS, acne inversa) is a chronic, painful, recurrent, and progressive inflammatory skin disease of the pilosebaceous unit. It is often characterized by the presence of nodules, abscesses, fistulas, and scars, mostly affecting intertriginous regions of the body, such as axillae, groins, gluteal, and submammary areas [1]. Acne inversa typically begins in early adulthood and affects both men and women, with a ratio of 1:3. The pathogenesis of HS is believed to be multifaced with components such as genetics, lifestyle, environment, hormones, and microbiota all taking part in this process. They cause immune activation around the terminal hair follicles and thickening of the horny layer, called hyperkeratosis, of the infundibulum, which most often occurs in the intertriginous regions of the body. Although acne inversa is not referred to as an infectious skin disorder, transmission of the bacteria in the intertriginous regions, where the skin folds tend to touch and rub, has the ability to trigger immune activation. Cells of both the innate and adaptive immune system secrete pro-inflammatory cytokines, such as TNF, IL-1β, and IL-17, to activate the tissue cells, which leads to immune cell infiltration and further inflammation. Eventually, this results in pus formation, tissue degradation, and scar development in patients with HS. Several studies brought evidence for immune dysregulation in acne inversa, however, the process of the immune response is still not fully understood.

Microbiome is defined as the total of microorganisms (such as fungi, bacteria, and viruses), their genomes, and interactions in a particular environment [2]. Recently, an interest in the microbiome of cutaneous diseases has increased tremendously. Of particular interest is the gut-brain-skin axis proposed by Stokes and Pillsbury in 1930, hypothesizing that emotional states could possibly alter the gut microbiome, increase intestinal permeability, and lead to systemic inflammation, and thus aggravate skin conditions. The microbiome has been suggested in the pathogenesis of hidradenitis suppurativa, however the link between the commensals and the host is yet to be established.

In this review, we aim to present the latest reports in the field of research on skin, blood, and gut microbiome in terms of hidradenitis suppurativa.

## 2. Skin Microbiome in HS

Several studies have tried to characterize the skin microbiome in hidradenitis suppurativa. In 2020, Riverain-Gillet et al. [3] studied the microbiome of clinically unaffected sites in 60 patients with HS, and 17 healthy controls. For bacterial cultures, a 5-cm^2^ skin surface was sampled for 30 s using a sterile E-swab. A total of 192 swab samples were analyzed and among these, 116 randomly selected samples were examined by 16S (V1–V2) rRNA gene amplicon sequencing. In terms of bacterial richness, it was similar between the HS skin and the skin of healthy controls. The authors have also found that skin of HS patients was characterized by an increased abundance of anaerobes, mostly *Prevotella*, as well as *Actinomyces*, *Mobiluncus*, and *Campylobacter ureolyticus.* In contrast, a lower abundance of skin commensals, such as *Staphylococcus epidermidis* and *Staphylococcus hominis,* was observed. The findings suggest that bacterial dysbiosis may be an event prior to lesion formation in HS.

Another research using swab samples was performed by Ring et al. [4] The authors investigated the bacterial composition of the luminal material found in HS tunnels with the use of NGS (next generation sequencing). This study enrolled 32 HS patients with tunnels that were present either in the groin (*n* = 17) or in the axilla (*n* = 15). A swab sample of the luminal material was taken during deroofing of the tunnels. The samples were then investigated using NGS targeting 16S (V3–V4) rRNA. Five microbiome types were identified in the material: *Porphyromonas* spp., *Corynebacterium* spp., *Staphylococcus* spp., *Prevotella* spp., and *Acinetobacter* spp. The study revealed a potential association between the presence of certain anaerobic bacteria, such as *Porphyromonas* spp. or *Prevotella* spp., and tunnels of HS patients. What is more, the two genera have previously been described in early and suppurating lesions, suggesting that *Porphyromonas* spp. and *Prevotella* spp. might be associated with the pathogenesis of HS.

In a case-controlled study that enrolled 30 subjects with HS and 24 healthy controls, the authors obtained punch biopsy specimens in order to perform a NGS targeting 16S and 18S (V3–V4) rRNA [5]. In HS patients, biopsy specimens were obtained from non-lesional skin and lesional skin from axilla or groin, whereas in healthy controls from the axilla only. In lesional skin, identified microbiome types consisted of *Corynebacterium* spp., *Porphyromonas*, and *Peptoniphilus species* spp. On the contrary, non-lesional skin was dominated by *Acinetobacter* and *Moraxella* spp. In healthy controls, *Porphyromonas* and *Peptoniphilus* spp. were not detected. Furthermore, the authors found no significant correlation between the number of species and the duration of lesions or its diameter. No difference in richness between the three groups was observed.

In a letter to the editor, Schneider et al. presented interesting results of their study [6]. The authors enrolled 11 HS patients and 10 healthy controls, both males and females, aged 19–61 years. Cyanoacrylate follicular biopsy (glue-based) and swab samples were collected from the axilla and groin area for each participant. In HS patients, both non-lesional and lesional skin was sampled. The 16S rRNA V3–V4 region was sequenced. The authors also used a metagenomic prediction tool (PICRUSt, Phylogenetic Investigation of Communities by Reconstruction of Unobserved States [7]) that allows users to predict metagenome functional content from marker genes. No significant difference in α-diversity was observed, however, a significant loss of β-diversity was noticed in both non-lesional and lesional skin compared to normal skin. No difference was found in bacterial composition between non-lesional and lesional skin. *Cutibacterium* was more abundant in normal skin (18.8% vs. 1%), and *Peptoniphilus* and *Porphyromonas* were more abundant in HS skin. Interestingly, in HS patients, a trend in altered β-diversity emerged between smokers and non-smokers, as well as between alcohol users and non-users, which suggests that lifestyle factors may impact skin microbiome diversity.

Encouraged by the results of the above-mentioned study, Ring et al. also performed their analysis [8]. The research group included the data from their previously published case-controlled study [5]. The study proved significant systematic differences between the groups (lesional vs. non-lesional; lesional vs. healthy controls). On the other hand, the non-lesional and healthy control samples were only moderately different. What is more, the authors found 420 differentially abundant genes between the lesional samples and healthy controls and 1120 between the lesional and non-lesional. Between the non-lesional and healthy controls, only eight genes were distinguished. The study showed that PICRUSt data identified cell growth and division (DNA replication, cell cycle-caulobacter, nucleotide excision repair, and mismatch repair) as strongly associated with lesional samples. The analyses of the two above-mentioned independent HS datasets provide great evidence that the key pathways involved in metabolism and genetic information processing are impacted in the HS skin microbiome. Overall, increased activity in these pathways may suggest increased microbial proliferation and turnover. The results of these studies give great potential for other researchers to investigate this topic further.

A few studies tried to bring up the topic of correlations between alterations in microbial dysbiosis and severity of HS. In research by Guet-Revillet et al. [9], samples of the pus from draining lesions (swabs) and non-draining lesions (punch biopsy or needle aspiration) of 65 HS patients underwent a 16S (V1–V2) rRNA sequencing. The samples were further compared to the microbial composition of clinically unaffected skin folds from the same participants. The study revealed an increased abundance of *Prevotella* and *Porphyromonas* spp. in HS lesional skin. Moreover, *Fusobacterium*, *Parvimonas*, *Streptococcus*, and bacteria of the *Clostridiales* order were the most abundant in patients with Hurley stage III disease severity. Similarly, Naik et al. also tried to investigate the above-mentioned correlation [10]. Samples of 12 enrolled HS patients and 5 healthy controls were taken from the axilla, gluteal crease, inguinal crease, and submammary fold and analyzed via 16S (V1–V3) rRNA sequencing. The study revealed an increased abundance of Gram-positive and -negative anaerobes and a decreased abundance of *Cutibacterium* spp. These findings prompted the authors to conclude that skin bacterial communities in HS are divergent compared to controls, with a greater disease severity associated with increased skin bacterial perturbations in HS patients.

Across all studies, the increased abundance of *Porphyromonas*, *Peptoniphilus,* and *Prevotella* spp. and a loss of skin commensal species, such as *Cutibacterium,* in HS lesions has been a consistent finding. Moreover, the two major bacterial skin species, *Staphylococcus aureus* and *Streptococcus pyogenes*, do not seem to contribute to acne inversa. Interestingly, chronic HS lesions were shown to contain bacterial biofilms as compared with skin of healthy controls from corresponding areas. Less biofilm was observed in clinically uninvolved skin in matched areas [11,12,13]. Even though bacterial dysbiosis in HS skin is a well-known fact, it remains unclear how the microbial dysbiosis affects HS severity, thus, further research in this area is expected. A summary of significant clinical studies concerning the skin microbiome in HS is presented in Table 1.

### The Role of Skin Microbiome in HS

The skin is the largest organ in the human body. Its main function is to act as a physical barrier against the external environment. The skin has its own ecosystem, which is made up of a large number of microorganisms, including bacteria, fungi, and viruses. Four main bacterial phyla can be distinguished in the skin microbiome: *Acinetobacteria*, *Bacteroidetes*, *Firmicutes*, and *Proteobacteria*. According to literature, the main link between the microbial molecules and the host happens through recognition of the microbial-associated molecular patterns (MAMPs) by the pattern recognition receptors (PRRs; like toll-like receptors (TLR) or NOD-like receptors (NLR)) on macrophages [14]. Moreover, bacteria seem to be involved in a well-established mechanism promoting HS lesion formation. In light of the research, mechanical friction, occurring within the intertriginous skin regions, leads to a local cell damage, resulting in the production of cellular damage-associated molecular patterns (DAMPs) and entering of bacterial components into the skin. This results in the activation of local resident immune cells, mainly macrophages, which secrete pro-inflammatory cytokines such as IL-1β and TNF (tumor necrosis factor) as a result of PRRs stimulation by DAMPs [15]. These mediators induce hyperplasia and hyperkeratosis of the infundibular epithelium of the hair follicle, causing occlusion. This, in turn, enhances propagation of local bacteria and stasis within the hair follicle, causing its dilatation. At this point, the role of the inflammasome, which is required for the detection of DAMP and the subsequent post-translational activation of IL-1β and IL-18, is already present. Activation of the pathway of currently best known inflammasome-NLRP3 (NOD-like receptor family, pyrin domain-containing 3; a cytoplasmic high-molecular-weight protein platform) is enhanced in skin lesions of patients suffering from HS, leading to increased expression of several inflammatory mediators, such as interleukin IL-1β, IL-17, caspase-1, S100A8, and S100A9 [16,17].

Increased abundance of *Prevotella* and *Porphyromonas* spp. in the skin microbiome of HS patients may take part in the pathogenesis of the disease via upregulation of antimicrobial peptide (AMP) secretion. This, in turn, leads to an increase of keratinocyte proliferation and recruitment of neutrophils and macrophages, which results in follicular occlusion and an increase in TNFα and NF-κB levels [18]. What is more, increased abundance of *Prevotella* spp. has been demonstrated to trigger the T helper 17 (Th17) immune responses and activate Toll-like receptor 2 (TLR-2), leading to an increased production of IL-1 and IL-23, which are involved in the pathogenesis of hidradenitis suppurativa [19,20]. According to literature, the production of IL-1β by keratinocytes while the microbial products are present increases the inflammatory state of HS lesions [21]. Additionally, it is worth mentioning that bacteria, owing to their ability to initiate a host inflammatory response, are relevant not only in lesional HS skin but also in non-lesional HS-prone skin, regardless of infection [22]. However, regardless of the research already conducted, it remains unclear whether skin microbiome dysbiosis triggers the process of HS lesion formation or is a consequence of the primary inflammatory process. Moreover, biofilm and its role in the pathogenesis of HS are worth mentioning in this review. According to studies, HS is a disease characterized by biofilm formation, which may be a trigger in the organism–host response [11,12]. A typically non-virulent commensal in healthy skin, *S. epidermidis*, has been shown to have a tendency for biofilm production in both lesional and non-lesional skin in HS patients [23]. Given the antibiotic resistance, rifampicin has been shown to efficiently eliminate biofilm in tunnels of HS patients [24]. Interestingly, as compared to healthy controls and HS patients without biofilm, patients with HS and identified biofilms have been found to have an increased concentration of CD4+ cells. Thus, biofilm has been suggested to stimulate the production of regulatory T cells, which supports the idea of dysbiosis as a factor in preclinical HS lesions [25]. It seems justified to study the microbiome with the use of modern genomic techniques from the point of view of the subsequent application of targeted antibiotic therapy. This procedure was described by Sabine Duchatelet et al. as a case of a patient who suffered from chronic acne fulminans and severe hidradenitis suppurativa [26]. Both of the diseases were unresponsive to many previous treatments, including: isotretinoin; oral steroids for 4 months, combined first with isotretinoin for 2 months then with tetracycline for 2 months; zinc; nonsteroidal anti-inflammatory drugs; and rifampin-clindamycin combination, alone for 5 months, then associated with infliximab for 7 months and with adalimumab for 5 months. After the lesional skin microbiome was elucidated using NGS, the targeted antibiotics were used, resulting in remission of long-lasting and severe AF with HS.

## 3. Gut Microbiome in HS

A study by McCarthy et al. [27], presented and positively received at the European Academy of Dermatology and Venereology (EADV) symposium in 2021, found a difference between the gut microbiome of individuals with acne inversa and healthy controls. The study enrolled 59 HS patients who provided fecal samples, as well as nasal and skin swabs. The results were compared with a control group consisting of 30 participants who provided fecal samples and 20 healthy controls who provided nasal and skin swabs. Furthermore, the samples underwent bacterial 16S rRNA gene amplicon sequencing on total DNA. A marker of microbial species richness or variation within a sample, called α-diversity, was significantly lower in subjects with HS. The decrease was also observed for other metrics of α-diversity, including Shannon and phylogenetic diversity. In a comparison of global microbial composition in all the samples (β-diversity), a microbiome separation between the HS and healthy controls was observed in all metrics tested. Less clustering within the HS samples was also noted. The authors found that one of the greatest differences in relative abundance between patients with HS and healthy control microbiomes were elevated levels of *Ruminococcus gnavus* and *Clostridium ramosum*.

Furthermore, in 2020, a case series by Kam et al. evaluated fecal samples of three patients with Hurley stage II or III. The samples underwent bacterial 16S rRNA sequencing. The study revealed that acne inversa may be associated with decreased gut microbiota species diversity, increased abundance of *Bilophila* and *Holdemania*, and reduced abundance of protective *Lachnobacterium* and *Veillonella*. Furthermore, the phylum *Firmicutes* was noted to be reduced in the HS group as compared to controls. Nonetheless, the authors underlined that, according to studies, smoking can reduce the relative abundance of *Firmicutes* in the intestine. Although very promising, the results came from a limited group of patients included in the observations [28].

On the other hand, a study by Lam et al. [29], which enrolled 17 HS patients and 20 healthy participants for 16S rRNA sequencing, found no differences between HS patients and controls in bacterial richness, Shannon, and inverse Simpson indices, nor in bacterial community structure based on Bray-Curtis or Jaccard metrics. What is more, no significant differences in α-and β-diversity were detected when stratified by BMI or smoking status. Nonetheless, LEfSe (linear discriminant analysis effect size; determines the features that are statistically different among biological classes) analysis indicated considerable taxonomic differences between stools of HS patients and healthy controls. Interestingly, the presence of Robinsoniella in the feces of the majority of HS patients and none of the healthy controls was proved.

Research investigating the role of the gut microbiome in hidradenitis suppurativa is ongoing. According to reports, the main link with the intestine is based on the increased risk of developing Crohn’s disease and ulcerative colitis [30]. Furthermore, both diseases respond to anti-TNF therapies, suggesting similar inflammatory pathomechanisms. With the growing understanding of the role of the gut microbiome in the disease, therapies to modulate the immune system using microbes are a promising new avenue of research. A summary of clinical studies concerning the intestinal microbiome in HS are presented in Table 2.

### The Role of Gut Microbiome in HS

The human gut microbiome is defined as a complex ecosystem consisting of bacteria, fungi, and viruses, all taking part in its host’s health [31]. It is known to be involved in a variety of processes, including digestion, synthesizing nutrients, and stimulation of the immune system [32,33]. For the most part, six phyla of the bacterial species can be found in the human gut microbiome: *Actinobacteria*, *Bacteroides*, *Firmicutes*, *Fusobacteria*, *Proteobacteria*, and *Verrucomicrobia* [34]. Multiple factors, including the type of infant delivery and feeding, diet, BMI, sex, antibiotic intake, the aging process, and stress, are able to influence the gut microbiome [35]. In healthy adults, the stool composition is stable over time. According to research, alteration of the gut microbiota composition can lead to multiple diseases, thus, it is important to keep intestinal eubiosis. As reported by Van Tongeren et al., decreased microbial diversity is associated with increased weakness and decreased health parameters, as well as increased inflammatory markers, such as TNF-α, IL-6, IL-8, and C-reactive protein [36].

As dysbiosis in the gut microbiome has been associated with multiple pathophysiological processes involving immune dysregulation, the hypothesis has been given that it could play a role in hidradenitis suppurativa. According to literature data, intestinal dysbiosis promotes the production of pro-inflammatory cytokines, such as IL-1β, IL-6, IL-17, and TNF-α, by the intestinal epithelia, which is followed by an increase in circulating inflammatory cytokines, IFN-γ, and TNF-α. This, in turn, leads to an increased level of matrix metalloproteinases (MMP-2, MMP-8, and MMP-9) and, subsequently, HS lesion formation [37].

What is more, as stated by McCarthy et al. [27], *R. gnavus* has been found to be overrepresented not only in HS participants of their study, but also in patients with Crohn’s disease, and is associated with spondylarthritis and irritable bowel syndrome (IBS). Its role in Crohn’s disease has been supported by the production of a proinflammatory polysaccharide, which increases the production of TNF-α by interacting with the toll-like receptor 4 (TLR4) of immune cells (e.g., dendritic cells) [38]. Thus, the authors postulated that the production of this polysaccharide may contribute to the pathogenesis of hidradenitis suppurativa. Consequently, it is possible that the comorbidities associated with HS have a common etiology, indirectly due to the activity of *R. gnavus*.

Interestingly, patients with HS have been shown to have low vitamin D levels, which seems to be correlated with disease severity [39,40,41]. By controlling proliferation and differentiation of epidermis and its structures (hair follicle in particular), vitamin D is able to regulate skin homeostasis [42]. Furthermore, at the skin level, vitamin D is known to take part in regulation of the immune response. According to studies, the intestinal microbiome might regulate vitamin D metabolism, thus, this pathway might play a significant role in signaling mechanism between the host and the gut microbiome [43,44]. Thus, it might be speculated that low vitamin D levels noted in HS patients could be a consequence of their intestinal dysbiosis, however, further research is highly needed.

## 4. Blood Microbiome in HS

Although the role of skin and gut microbiome has been increasingly established and studied in HS patients, the results of only two published studies on blood microbiome in these patients are contradictory, hence knowledge is very limited in this case [45,46]. Patricia Hispán et al. performed a single-center case-control study to assess the presence of BactDNA in the peripheral blood of patients with active HS. The study included 50 HS patients and 50 healthy controls, from whom peripheral blood samples were collected. The DNA of bacteria was significantly more likely to be detected in HS patients (34%) vs. controls (2%). The most frequently detected were Gram-negative bacteria, especially *Escherichia coli*, which is evaluated as a member of the intestinal microbiome of over 90% of healthy individuals [47]. Thus, authors hypothesized the pathogenesis of HS may be affected by bacterial translocation from the intestinal lumen, as it was suggested in psoriasis and inflammatory bowel disease [45,48,49]. The fact worth underlining is that the authors did not perform bacterial cultures from blood in these patients. The second study, carried out by Hans Christian Ring et al., was based on blood investigation from 27 HS patients and 26 healthy controls using two methods: next generation 16S ribosomal RNA gene sequencing (NGS), as well as routine anaerobic and aerobic blood culturing. Blood culturing samples were negative, however, quantities of several bacteria, including *Acinetobacter* and *Moraxella*, were found in the NGS samples. According to the authors, the differences in the results between the two used methods were caused by the high sensitivity of NGS 12 compared to our traditional cultures [46,50]. Despite the differences in methodologies, HS patients’ peripheral blood investigated by NGS did not differ in bacterial composition from that of healthy controls [46]. A summary of clinical studies concerning the blood microbiome in HS are presented in Table 3.

### The Role of Blood Microbiome in HS

In view of the lack of studies investigating the blood microbiome in hidradenitis suppurativa, there are still no strict suggestions about its role played in the course of the disease. It may be hypothesized, based on the studies performed on the other clinical conditions, that the blood cytokine response may be significantly influenced by the presence of bactDNA in serum [49]. Further research is needed to investigate the role of blood microbiome in HS.

## 5. Lifestyle, Probiotics, and Dietary Approaches in HS

A well-known fact is the influence of lifestyle on the course of HS. Modifications such as weight loss, smoking cessation, and diet restrictions are recommended for patients with HS, as there is some evidence that they have the ability to improve the disease [51]. Moreover, the above-mentioned factors can also influence the microbial composition [52,53]. As research has shown, *Proteobacteria* and *Bacteroidetes* phyla tend to be increased in the gut microbiome in smokers. On the other hand, *Actinobacteria* and *Firmicutes* phyla have been found to be decreased [54]. Smoking can also lower the diversity of the gut microbiome. There are several proposed mechanisms that could explain the effects of smoking on intestinal microbiome, including the changes in acid-base balance, enhancement of oxidative stress, and alterations of intestinal tight junctions and intestinal mucin composition. Hence, smoking cessation could lead to a restoration of the normal intestinal flora.

According to the World Health Organization (WHO) and the Food and Agriculture Organization of the United Nations (FAO), probiotics are defined as live microorganisms that confer health benefits upon the host when administrated in adequate amounts [55]. As proposed by Ring et al., probiotics are considered as a promising option in supporting HS treatment given that they can restore a skin bacterial homeostasis by replenishing the abundance of *Cutibacterium* spp., *Corynebacterium*, and *Staphylococcus* [56]. Highlighted by the authors, in order to achieve the potential prophylactic efficacy, the application of probiotics should be focused mainly on the prelesional skin. Furthermore, oral probiotics also deserve some attention. Probiotics are generally considered safe for humans with limited side effects. They are able to control the functioning of the gut microbiota by changing its population. The exact mechanisms by which the gut microbiome influences the course of the disease have multiple implementations to the skin. Commensals in the intestine have the ability to influence systemic inflammation, oxidative stress, pathogenic bacteria, glycemic control, and tissue lipid content [57]. Therefore, by modulating the intestinal microbiome, oral probiotics can have an influence on skin diseases. When it comes to dermatoses, atopic dermatitis (AD) is the most well-studied dermatological condition for the use of such therapy [58], nonetheless, the use of probiotics has also been explored in acne vulgaris, a common disease of the pilosebaceous unit, as well as psoriasis [59]. More research on the potential use of probiotic bacteria species and their strains is highly needed for a better understating of its role in the prevention and treatment of multiple diseases. Remarkable knowledge gaps exist in this very promising and significant area of research, mainly due to the heterogeneity between studies and variability in the probiotic strains utilized. Given the common factor between hidradenitis suppurativa and the above-mentioned diseases, the inflammation, the probiotics supplementation in HS appears to be warranted. These findings may help with the development of the most up-to-date therapeutic strategy, which is personalized therapy.

What is more, as suggested in several studies, diet can also influence the course of the disease. For instance, avoiding a high-fat diet is proposed as a great secondary preventive measure for acne inversa [60]. There are also some reports supporting limiting simple carbohydrate and sugar intake, eliminating dairy products, as well as avoiding foods that contain brewer’s yeast [61]. Diet modifications could be beneficial to some HS patients, nonetheless, it has not been fully explored whether diet may prove to be valuable in limiting the HS severity. Further research in this area is highly needed to determine the potential of such modalities.

## 6. Material and Methods

The presented study is a narrative review. The PubMed database has been searched for articles relevant to this review. The analysis reviewed the papers published by July 2022. Search terms included “hidradenitis suppurativa” or “acne inversa” and “microbiome” or “biofilm” or “bacteria”. Duplicate articles, and those written in languages other than English, were excluded. The obtained articles were analyzed via content and quality analysis and then compared.

## 7. Conclusions

Hidradenitis suppurativa is a complex disease, pathogenesis of which cannot be simplified to microbiome disturbance only. Even though HS is not considered as a primary infectious disease, it is a dermatosis that predisposes patients to develop autoinfection or inflammatory skin lesions. Nevertheless, carrying out further research considering the role of the microbiome, particularly intestinal, in hidradenitis suppurativa is required, as the microbiome composition may differ from healthy controls significantly, especially taking into account that patients with HS are often smokers, have an elevated BMI, and are exposed to a long-term antibiotic therapy, which may affect the composition of their microbiome. Further research will not only determine the role of microbiome dysbiosis in the pathogenesis and progression of the disease, it will also identify novel therapeutic targets, which is of the utmost importance.

## Figures and Tables

**Table 1:** A summary of significant clinical studies concerning the skin microbiome in HS.

Authors	Analyzed Sample	Analysis Method	Study Results
Riverain-Gillet, É. et al. [3]	Skin swab	16S rRNA gene sequencing (V1–V2)	↑*Prevotella*, ↑*Actinomyces*, ↑*Mobiluncus*, *Campylobacter ureolyticus*, ↓*Staphylococcus epidermidis*, ↓*Staphylococcus hominis*
Ring, H. C. et al. [4]	Swab of the luminal material found in HS tunnels	16S rRNA gene sequencing (V3–V4)	↑*Prevotella*, ↑*Prophyromonas*
Ring, H. C. et al. [5]	Skin biopsy	16S and 18S rRNA gene sequencing (V3–V4)	In lesional skin, ↑*Corynebacterium* spp., ↑*Porphyromonas*, ↑*Peptoniphilus species* spp. In non-lesional ↑*Acinetobacter* and ↑*Moraxella* spp.
Guet-Revillet, H. et al. [9]	Swab samples of the pus from draining lesions and punch biopsy or needleaspiration of non-draininglesions	16S rRNA gene sequencing (V1–V2)	In lesional skin, ↑*Prevotella*, ↑*Prophyromonas*
Naik, H. B. et al. [10]	Skin swab	16S rRNA gene sequencing (V1–V3)	↑*Porphyromonadaceae*, ↑*Prevotellaceae families*, ↑*Fusobacteria phylum*, ↓*Cutibacterium* spp.

**Table 2:** A summary of clinical studies concerning the intestinal microbiome in HS.

Authors	Analyzed sample	Analysis Method	Study Results
McCarthy, S. et al. [27]	Fecal sample	16S rRNA gene sequencing	↑*Ruminococcus gnavus*, ↑*Clostridium ramosum*
Kam, S. et al. [28]	Fecal sample	16S rRNA gene sequencing	↑*Bilophila*, ↓*Lachnobacterium*
Lam, S. Y. et al. [29]	Fecal sample	16S rRNA gene sequencing	The presence of *Robinsoniella* in the feces of the majority of HS patients and none of the healthy controls

**Table 3:** A summary of clinical studies concerning the blood microbiome in HS.

Authors	Analyzed sample	Analysis Method	Study Results
Ring, H. C. et al. [46]	Blood sample	16S rRNA gene sequencing (V3-V4), anaerobic and aerobic blood culturing	No difference in bacterial composition in peripheral blood between HS patients and healthy controls
Hispán, P. et al. [45]	Blood sample	16S rRNA gene sequencing	↑Presence of bacterial DNA in HS patients vs. healthy controls, ↑*E. coli.*
